# Autonomously Propelled Colloids for Penetration and Payload Delivery in Complex Extracellular Matrices

**DOI:** 10.3390/mi12101216

**Published:** 2021-10-06

**Authors:** Shrishti Singh, Jeffrey L. Moran

**Affiliations:** 1 Department of Bioengineering, George Mason University, Fairfax, VA 22030, USA; ssingh64@gmu.edu; 2 Department of Mechanical Engineering, George Mason University, Fairfax, VA 22030, USA

**Keywords:** extracellular matrix, drug delivery, self-propelled particles, ECM, active colloids

## Abstract

For effective treatment of diseases such as cancer or fibrosis, it is essential to deliver therapeutic agents such as drugs to the diseased tissue, but these diseased sites are surrounded by a dense network of fibers, cells, and proteins known as the extracellular matrix (ECM). The ECM forms a barrier between the diseased cells and blood circulation, the main route of administration of most drug delivery nanoparticles. Hence, a stiff ECM impedes drug delivery by limiting the transport of drugs to the diseased tissue. The use of self-propelled particles (SPPs) that can move in a directional manner with the application of physical or chemical forces can help in increasing the drug delivery efficiency. Here, we provide a comprehensive look at the current ECM models in use to mimic the in vivo diseased states, the different types of SPPs that have been experimentally tested in these models, and suggest directions for future research toward clinical translation of SPPs in diverse biomedical settings.

## 1. Introduction

The extracellular matrix (ECM) is a complex three-dimensional network of proteins, sugars, cells, and biomolecules [1] that provides support to biological tissue and aids cellular processes [2] such as differentiation and signaling [3,4]. The ECM is a dynamic environment that often undergoes remodeling and plays an instructive role in regulating tissue homeostasis [5]. In diseases such as cancer [2,6] or fibrosis, which is implicated in over 45% of deaths in the developed world [7], the ECM undergoes abnormal growth or repair, which can cause it to become dense and stiff [8], forming a mechanical and biochemical barrier for drug delivery systems since the ECM limits the transport of drugs to diseased cells [9]. Such impediments to delivery are also seen in bacterial biofilms, which consist of bacteria residing within a matrix that resembles diseased ECMs, but with a different biochemical composition. Biofilms appear both in vivo and ex vivo and are associated with 80% of chronic infections in humans [10]. Therefore, new and innovative payload delivery methods are needed to address the challenge of penetrating the defenses of both tissue and bacterial ECMs.

**Figure 1:**
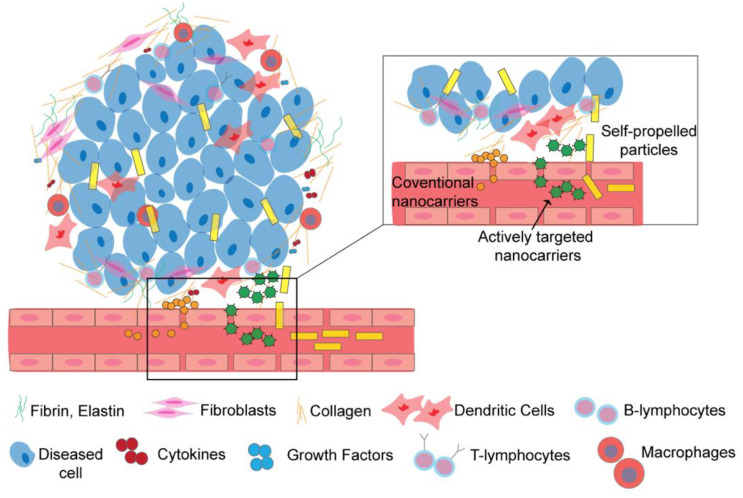
Vision of using self-propelled particles in ECM environments. The figure shows a schematic of the extracellular matrix surrounding a solid tumor and the movement of different types of drug delivery nanocarriers within it. Conventional nanocarriers with no active targeting moieties are stuck at the periphery of the dense microenvironment surrounding cells. Active nanocarriers (which are not able to propel themselves but are decorated with targeting moieties) move further into the ECM (inset) compared to passive nanocarriers but the ECM still acts as a barrier to them. Self-propelled particles (SPPs) can propel further in the ECM and deliver their cargo (e.g., drugs) closest to the diseased cells; as a result, SPPs are dispersed throughout the tumor microenvironment, dramatically improving the distribution of the therapeutic cargo within the tumor compared to passive nanocarriers.

For effective treatment of ECM-mediated diseases, such as different types of cancers [11] or idiopathic pulmonary fibrosis [12,13], it is important to deliver the drug to the diseased site and not the surrounding environment, for maximal therapeutic efficacy [14] and minimal side effects resulting from drug accumulation in healthy tissues. Better patient outcomes therefore necessitate improved penetration of drug delivery systems through the ECM [15]. Conventional nanocarriers, such as liposomes [16,17], polymeric nanoparticles [18,19], or metallic nanoparticles [20,21], rely on passive diffusion for transport through the ECM and so cannot penetrate diseased ECM effectively. Consequently, they frequently release their payload outside the disease site [22]. Due to limited diffusional transport of the payload in the ECM [23,24], and other factors such as renal, hepatic, or immune clearance, conventional drug delivery nanocarriers have a <1% delivery efficiency to tumors [25,26]. Active drug delivery nanocarriers with attached ligands [27], such as antibodies [28,29], peptides [30,31], or small molecules [32], which target specific receptors on a diseased cell or in the ECM, have shown promise in clinical and pre-clinical trials to increase the efficiency of drug delivery. However, these actively targeted nanocarriers need to be in the vicinity of their target receptor to recognize it for maximum therapeutic benefit [33], which remains a challenge to the development of these nanocarriers [34]. These challenges motivate the need for drug delivery systems that can autonomously move through the dense, stiff ECM and deliver therapeutic payloads (e.g., chemotherapeutics, antifibrotics, or antibiotics) in the closest neighborhood of the diseased cells. Figure 1 shows a schematic of the ECM, including several of the important cellular and non-cellular constituents. The figure demonstrates that SPPs have the potential to dramatically enhance the distribution of therapeutic molecules in diseased ECM environments, which could lead to revolutionary improvements in patient outcomes resulting from higher drug efficacy, lower doses, and reduced side effects. 

One potential way to realize this vision is to use self-propelled particles (SPPs) as delivery vehicles. First demonstrated in 2004 in the form of hydrogen-peroxide-powered platinum/gold nanorods [35], SPPs are active colloidal particles that typically range in size from 30 nm to 30 µm [36,37] and can convert physical (e.g., magnetic [38,39] or electric fields [40,41] ), ultrasound [42,43,44], or chemical (e.g., hydrogen peroxide [35] and enzymatic reactions [45]) energy into motion. In addition to autonomous movement, it is well-known that SPPs can carry and deliver payloads to specific locations [46]; hence, they have been widely considered for drug delivery applications since 2010 ((if not earlier [47]). However, since most fluids and tissues in the body are viscoelastic [48,49] and/or non-Newtonian, it is crucial that candidate SPP designs be tested in media that exhibit these same characteristics. More recent studies [50,51,52] have demonstrated the motion of SPPs in hydrogels that resemble the ECM, to potentially exploit them as drug delivery systems in tissues as well as the gastrointestinal tract [53]. Currently, many research studies are delving deeper into improving the motion capabilities of SPPs in viscoelastic media that resemble ECM. Since the ECM is often dense, thick, and viscoelastic [49] (especially in disease states), designing SPPs for navigation in ECM both poses additional challenges and opens new opportunities for innovation.

In this review, we examine recent research efforts to use SPPs to penetrate extracellular matrices. Section 2 provides a brief introduction into the role of ECM as a barrier to drug delivery and describes the types of artificial and naturally derived ECM models that have been studied. Section 3 describes the various SPP designs (including various propulsion strategies and energy sources) and the results showing their motion through different types of ECM. Our primary focus is on experimental demonstrations of SPP designs that have been shown to effectively move in models of the ECM; however, theoretical investigations ultimately have the potential to inform the design of future microswimmers, a topic we revisit in Section 4, which identifies open questions and suggests future research directions.

## 2. Biomimetic Models of Extracellular Matrix

Researchers have used a variety of *in vitro* platforms to explore the use of autonomously propelled particles to penetrate ECM. These materials generally fall into two categories: naturally derived and synthetic. Each has advantages and disadvantages. For example, naturally derived materials retain the original protein composition of in vivo ECM, and thus faithfully represent the biochemical cues present in real tissues. On the other hand, synthetic materials tend to be more easily tunable, enabling independent variation of a variety of mechanical and biochemical properties [54]. As a first step towards the goal of using SPPs to penetrate real tissues, researchers have made use of both synthetic and naturally derived ECMs to demonstrate the efficacy of various SPP designs. 

### 2.1. Naturally Derived ECM

#### 2.1.1. Matrigel

Matrigel is the trade name of an ECM derived from a murine sarcoma known as Engelbreth–Holm–Swarm (EHS), dating back 40 years. These tumor extracts contain large quantities of ECM proteins [55] and are developed and marketed as Matrigel or EHS matrix [56]. Matrigel is composed of four major ECM proteins: laminin, collagen IV, entactin, and heparin sulfate proteoglycan perlecan [57]. The percentage composition of laminin and collagen in Matrigel are ~60% and ~30%, respectively. Entactin makes up ~8% whereas ~2–3% of Matrigel is composed of heparin sulfate proteoglycan perlecan [57]. Collagen IV is the most abundant type of collagen in Matrigel. Others include collagen I, XVIII, VI, and III [58]. The most dominant isoforms of laminin in the Matrigel are α1, β1, and γ1, which make up the heterotrimer laminin 1 [58,59]. Multiple adhesion sites are available on laminin 1 for the attachment of different cell types, such as epithelial, endothelial, and tumor cells [60]. Moreover, laminin-1 peptides promote tumor growth and metastasis [61,62]. Matrigel also contains growth factors and tumor-derived proteins, such as Transforming Growth Factor (TGFs) [63] and Fibroblast Growth Factors (FGFs) [64], along with enzymes, such as matrix metalloproteinases (MMPs) [56,65], which cleave ECM proteins such as collagen and cause reconstruction of the ECM. Because of the quantity and variety of proteins and biomolecules present, Matrigel exhibits some viscoelastic properties [66], mimicking the behavior observed in real tumor tissues.

Matrigel undergoes gelation to create a hydrogel at temperatures in the range 22–37 °C in which enactin acts as a crosslinker between collagen IV and laminin. Based on the total concentration of ECM protein in Matrigel formulations, Matrigel is used for different applications. Low protein concentration Matrigel is used to culture cells such as cardiomyocytes [67,68] and human pluripotent stem cells (hPSCs) [69]. Higher protein concentrations of Matrigel have been used as 3D constructs to encapsulate cells [70,71] and for organoid assembly [72,73]. Since the mechanical properties of the media influence the propulsion of SPPs, it is important to know the mechanical properties of Matrigel, in addition to its biological components. Since Matrigel is a versatile environment and has variability in its composition, previous studies [74,75] have reported shear modulus values of the range of 34–55 Pa. Rheology studies by Soofi et al. using AFM indentation [76] have shown the average elastic modulus of Matrigel to be 440 ± 250 Pa. Another study by Reed et al. [77] reported the elastic modulus of Matrigel films after a gelation time of 1 h as 650 Pa. By comparison, the elastic modulus of invasive ductal carcinoma was previously measured by Miura et al. to be 22.25 kPa [78]. Thus, although it is convenient and recapitulates the biochemical ECM microenvironment often encountered in vivo, Matrigel does not always accurately replicate the mechanical properties of in vivo tissue.

#### 2.1.2. ECM Derived from Decellularized Tissues (DT)

ECM can also be derived from decellularized tissues (DT) wherein the cellular components of the tissue are removed through chemical or enzymatic [1,79], or mechanical disruption [80]. The non-cellular component of the tissue is used as ECM. ECM hydrogels from DT have been obtained from almost every organ system. The first ECM was obtained from decellularization of small interstitial submucosa in 1973 [81]. Since then, various tissues, such as pancreas [82], urinary bladder [83,84], lung [85], and others, have been used to create ECM-derived hydrogels. The mechanical properties of ECM obtained from decellularized matrix have been studied for decellularized cardiac tissue (C-ECM). Based on the conditions of decellularization, the compressive modulus of C-ECM ranged from 5.8 to 2.4 kPa [86]. Rheology studies on ECM obtained from decellularized porcine liver (L-ECM) showed the elastic modulus of L-ECM ranges from 31.8 kPa to 5.7 kPa based on the concentration of collagen present [87]. Recently, it was shown that hydrogels derived from human lung tissues accurately replicated the Young’s modulus (as a measure of stiffness) of the original lungs, but exhibited greater stress relaxation (a measure of viscoelasticity) compared to the lungs [88]. Hence, naturally derived ECMs have great variability in their physical structures and mechanical properties, an important aspect in particle propulsion. Future studies should illuminate the effects of mechanical and viscoelastic properties on the propulsion of SPPs through the ECM. 

### 2.2. Hydrogels Mimicking ECM

In recent years, researchers have developed a wide variety of synthetic and natural biomaterials that serve as *in vitro* models of the extracellular matrix [89]. Since naturally derived ECM has variability in its physical properties, synthetic hydrogels that can be systematically modified to control their biochemical and mechanical properties can be used as a successful ECM mimic to study the propulsion of SPPs. 

#### 2.2.1. Hyaluronic Acid Hydrogels

Hyaluronic acid (HA) is a naturally occurring linear polysaccharide with repeating units of D-glucuronic acid and N-acetyl-D-glucosamine linked by glycosidic bonds [90], which is found in natural ECM as well as biofilms [91], and can be readily modified to create hydrogels. A study by Nimmo et al. [92] synthesized HA-PEG hydrogels with polyethylene glycol (PEG) being the crosslinker and characterized their mechanical properties. The elastic modulus of the HA-PEG gels with different crosslinker concentrations ranged from 2.75 ± 0.54 kPa to 6.79 ± 0.62 kPa, similar to the elastic moduli of brain and nerve tissues. A follow-up study by Owen et al. [93] increased the elastic modulus of HA hydrogels with a furan substitution. The elastic modulus of the HA gels increased to 16.12 ± 0.94 kPa due to increased crosslinking between the furan-substituted HA and maleimide PEG. The viscoelastic properties of the HA gels have been determined by studies from Borzacchiello et al. [94]. Their study showed that a higher concentration HA gel with high crosslinking density has an elastic modulus of 3.04 kPa and a loss modulus of 0.16 kPa, signifying that HA gels demonstrate viscoelastic behavior with comparable mechanical properties to collagen gels. Their study also demonstrated that the viscosity of HA gels was 485.20 Pa·s, comparable to those of the collagen networks found in ECM. Since HA is present both in many ECMs as well as some biofilms, and because of the adjustable nature of their mechanical and viscoelastic properties, HA-based hydrogels show promise as a tunable, biomimetic model of ECM for screening studies of novel therapeutic SPP designs.

#### 2.2.2. Mucin Gels

Mucins are a family of functional glycoproteins that assemble in vivo to form mucus, the viscoelastic and protective barrier lining various tracts, such as the gastrointestinal and respiratory tract [95]. Studies by Celli et al. [96,97] showed that mucin from porcine gut underwent drastic changes in its viscoelastic behavior at different pH levels, with the elastic modulus being more dominant than the loss modulus at a low pH. This indicated a gel-like material, whereas the opposite is true at pH greater than 6, which indicates a liquid-like material. This sol-gel transformation of mucin gels indicates a shear-thinning behavior, which has been studied by various other works [98,99,100]. Different works have reported characterizing the rheological properties of tracheal mucus at different frequencies. These studies revealed that the average elastic modulus of tracheal mucus in humans can range from 0.2 to 32 Pa at low frequencies and 10 to 52 Pa at higher frequencies [101,102,103]. A study by Bastholm et al. [104] studied the viscoelastic properties of cervical mucus gels that coat the cervical canal during pregnancy. The study calculated the elastic modulus of these gels as 19.5 Pa, with a loss modulus of 5.4 Pa. Hence, mucin gels can be a good representation of in vivo conditions and serve as a potential model for drug delivery using SPPs.

#### 2.2.3. Collagen Gels

Collagen gels have also been used as models of ECM in which SPPs have been tested. Ramos-Docampo et al. [105] demonstrated locomotion of manganese ferrite nanoparticles decorated with collagenase enzymes that break down collagen, a protein which is naturally abundant in the ECM (as discussed above). This approach not only makes use of readily available energy sources in the ECM, the SPPs also break down the ECM microstructure at the same time. This capability could be advantageous for SPP-mediated treatment of diseases in which the ECM is stiff and fibrotic, such as cancer or fibrosis. As discussed below, collagenase has also been patterned onto magnetic nanoparticles for the same purpose [106].

#### 2.2.4. Other Hydrogel ECM Mimics

The development of synthetic or naturally derived hydrogels to serve as *in vitro* mimics of ECM is a robust field of research [89,107], enabling both fundamental biological studies (e.g., of cell-ECM interactions) as well as applied studies developing hydrogels for tissue engineering, regenerative medicine, and drug delivery. In addition to the hydrogel types discussed above, a variety of other materials, such as alginate [108], polyethylene glycol [109], and gelatin methacryloyl (GelMA) [110], have been used to create synthetic hydrogels that mimic the ECM. However, since the focus of this review is on developing SPPs that penetrate ECM, in this section we have concentrated on synthetic hydrogels in which SPPs have been tested. 

### 2.3. Biofilms

Biofilms are multicellular communities of bacteria enclosed in a specialized ECM known as the extracellular polymeric substance (EPS). The EPS consists mostly of polysaccharides, DNA, proteins, and lipids [111]. The structural and biochemical components of EPS differ in important ways from the ECM in animal tissues. However, biofilms also share important similarities with the ECM in tissues. Both of these are three-dimensional polymeric networks that provide structural and biochemical support to cells. Both act as mechanical and biochemical barriers that protect problematic cells, whether they are cancerous cells in a solid tumor ECM or bacteria in an infectious biofilm. In both cases, the interactions between cells and their surrounding ECM affect the cells’ phenotype and promote their resistance to treatments. For example, the tumor ECM provides protection to malignant cells and can confer resistance to chemotherapeutics [112]; by the same token, bacteria in biofilms are typically more resistant to antibiotics [111] than the same bacteria would be in a planktonic state. Thus, like the ECM in diseased tissues, effective remediation of biofilms demands that we develop abilities to actively penetrate the EPS that surrounds bacteria and protects them from the outside world.

## 3. Self-Propelled Particles Movement in ECM

Most tissues in the body exhibit viscoelasticity, meaning they show a time-dependent response to deformation and dissipate some of the energy that was expended to accomplish the deformation [49]. A growing body of evidence indicates that ECM viscoelasticity influences fundamental cell processes such as differentiation, spreading, migration, and ECM synthesis, and plays a key role in the procession of diseases such as fibrosis and cancer [49]. Considering the growing body of literature on microscale self-propulsion in shear-thinning [113,114,115] and viscoelastic media [116,117], it is reasonable to expect that ECM viscoelasticity will influence the efficiency of the self-propulsion. Accordingly, to develop SPPs that are attractive for biomedical applications in drug delivery in human tissues, it is critical that the swimming performance of SPPs be tested in environments that possess similar viscoelastic characteristics to in vivo human tissues.

Most nanocarrier-based drug delivery systems enter the bloodstream and extravasate through tumor blood vessels to reach the tumor site; this effect is known as the Enhanced Permeability and Retention (EPR) effect [118,119]. The rate of accumulation of these systems at the tumor site is low, which significantly decreases the drug delivery efficiency [26]. Many studies have developed so-called “active targeting” drug delivery nanocarriers that are functionalized with targeting moieties to increase the effectiveness of delivery, but these are still plagued by issues such as poor drug loading, rapid release of drugs, and difficulties in reaching the diseased site due to internal barriers such as the ECM. Therefore, SPPs are a potential method to resolve these issues by reaching in the vicinity of the tumor or diseased site after traversing the ECM. In this section, we review recent efforts to use SPPs to penetrate ECMs of various types outlined in Section 2. 

Table 1 summarizes the different types of SPPs that have been tested in ECM models to date, including a comparison of their design, motion mechanism, advantages, and disadvantages.

### 3.1. Use of Physical Forces for Movement of SPPs in Hydrogels

Magnetic fields and ultrasound are regularly used in a variety of clinical applications, from MRI to photoacoustic computed tomography. Both can also be used for propulsion of SPPs. Herein, we refer to these as “physical” forces to distinguish them from chemically powered SPPs (note that although these particles rely on external fields for propulsion, they are still commonly referred to as SPPs because, like other SPP designs, they convert ambient energy into motion). Movement of SPPs using external physical forces has been demonstrated in both natural and synthetic hydrogels resembling ECM. 

#### 3.1.1. Magnetic Forces

**Figure 2:**
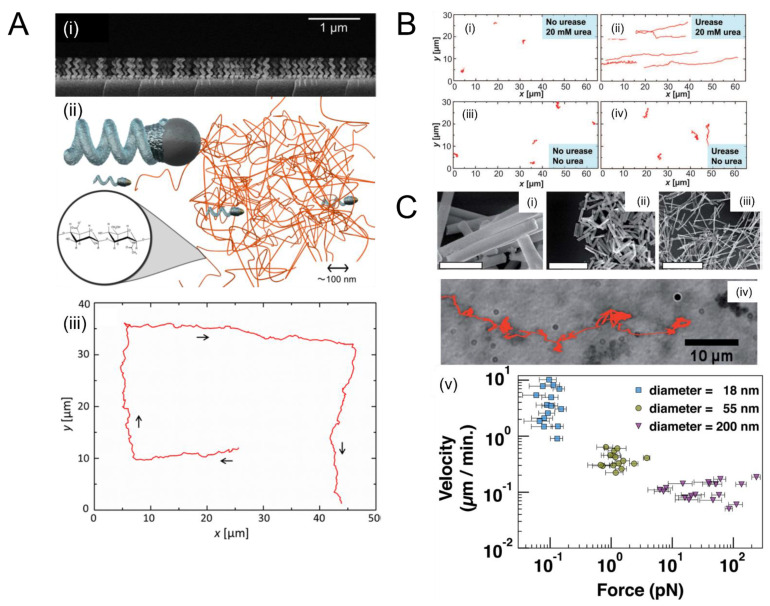
Magnetic propulsion of micro- and nanoparticles in model extracellular matrices. (**A**) (i) Scanning electron micrograph of magnetic helices synthesized using glancing angle deposition. (ii) Motion of micro- and nanohelices in hyaluronic acid (HA) gels. While microhelices encounter steric hindrance in the polymer mesh (top), nanohelices comparable to the mesh size move more efficiently (bottom). (iii) 2D trajectory of a nanoscale helix in HA gel showing controllable motion in all four directions. When the same helices were placed in water, the directionality was reduced (not shown) because, in the less-viscous aqueous environment, Brownian motion exerts a greater destabilizing influence on orientation. Panel (**A**) adapted with permission from [50] Copyright © 2021 American Chemical Society. (**B**) Trajectories of nanohelices (also synthesized using GLAD) through viscoelastic mucin gels. The nanohelices are decorated with urease enzymes that locally raise the pH, liquefying the gel and enabling efficient motion. Panel (ii) shows that motion is, by far, the most efficient in the presence of both urease enzymes and urea fuel, compared to cases when either is absent (i, iii, iv). Panel (**B**) adapted from [52] Copyright © 2021, the authors. (**C**) Cylindrical ferromagnetic nickel (Ni) nanorods were synthesized through templated electrodeposition in three different diameters: 200 nm (i), 55 nm (ii), and 18 nm (iii). Under an inhomogeneous magnetic field, the nanorods experience a force that depends on the product of the field strength and field gradient and move through Matrigel (a model of ECM) by magnetophoresis. (Panel iv shows the motion trace of 55-nm rods). (v) Thinner rods translate faster because they encounter less steric hindrance. However, they also carry less cargo than thicker rods (not shown). Panel (C) reproduced from [51]. © The Authors, some rights reserved; exclusive licensee AAAS. Distributed under a CC BY-NC 4.0 license.

External magnetic fields are common in medicine; for example, they are frequently used in clinical imaging tools such as MRI. Hence, the use of magnetic forces to propel SPPs in natural or synthetic matrices has been explored. Figure 2 depicts notable advances in the use of magnetic propulsion for SPP-mediated penetration of ECM models. One of the earliest studies by Kuhn et al. [106] demonstrated that 145 nm superparamagnetic (SPM) ferrous oxide spherical nanoparticles coated with polyethylene glycol (PEG) achieved a velocity of 0.42 ± 0.04 µm s^−1^ in Matrigel, which was seven times greater than same-sized silica coated nanoparticles when guided under an external magnetic field. A PEG coating was used to reduce non-specific (i.e., electrostatic and van der Waals) interactions between the ECM and the nanoparticles. Their study also demonstrated that PEG-coated SPM nanoparticles of 400 nm radius do not enter Matrigel, suggesting that steric effects significantly impede SPPs’ motion in natural matrices (a similar conclusion was reached by Mair and Superfine [51] in the context of cylindrical rods, as discussed below). Kuhn et al. followed up [120] on their previous study and surface-attached the enzyme collagenase to PEG-coated SPM nanoparticles. Collagenase is a proteolytic enzyme that degrades collagen, a common constituent in most natural matrices. By attaching collagenase to 145 nm SPM nanoparticles, the speed of the particles averaged 0.025 ± 0.01 µm s^−1^ in Matrigel supplemented with collagen (1:4 ratio of Matrigel to supplemented collagen). Although this speed was slower than in the previous one [106] (0.42 ± 0.04 µm s^−1^), the supplemental collagen caused a speed reduction; this observation illustrates that, as expected, SPPs move more slowly in denser ECM. The speed achieved by the nanoparticles was comparable to the literature values for the velocity of metastatic cells (0.02 ± 0.01 µm s^−1^) [121]. Hence, use of proteolytic enzymes that cleave the mesh-like structure of the ECM can provide a way for SPPs to be transported through a dense, fibrotic ECM when supplemented, especially in fibrotic states when collagen is often present in excess.

Another study by Mair and Superfine studied the propulsion of cylindrical nanorods in Matrigel [51] (Figure 2C). Their study showed that nickel nanorods with three different diameters (200, 55, and 18 nm) move in a static magnetic field generated by a permanent magnet. The length of the nanorods ranged from 1 to 2 µm and their surface was modified with PEG to reduce non-specific rod–ECM interactions. Under the same field conditions, thinner rods moved more rapidly (highest velocity attained by the 18-nm nanorods). The 55 and 200 nm diameter rods experienced significant steric hindrance due to their larger diameter. Thinner rods exhibit more variability in their orientation with respect to the direction of the magnetic field. These variations, which ultimately arise from Brownian fluctuations, could in turn help the rods evade steric hindrances due to the dense ECM mesh network, potentially improving their motion efficiency. However, translational speed is only one variable to consider when designing an SPP-based drug delivery system. Another important figure of merit is the amount of cargo transported per unit time. To this end, the authors calculated figures of merit for payload towing, assuming either a volumetric or surface-based loading of cargo. For volumetric loading, 200-nm-diameter rods performed best and 18-nm-diameter rods worst. For surface loading, the performance of the 18- and 200-nm rods were comparable and both exceeded those of the 55-nm rods. Hence, Mair and Superfine’s work emphasized the importance of size and shape in achieving useful transport velocities of SPPs in biological ECM mimics. Their work also underscores the importance of striking the right balance between making SPPs small enough to move efficiently through the porous ECM mesh network (favoring smaller particles) but large enough that they carry a therapeutically relevant amount of cargo (which favors larger particles). 

To demonstrate the movement of SPPs in viscoelastic ECM models, Schamel et al. [50] created nanoscale rigid helical particles from silica and demonstrated their propulsion in hyaluronic acid (HA) gels (Figure 2A). The nanohelices were produced via a physical vapor deposition process (PVD) known as glancing angle deposition (GLAD), in which material is deposited onto a regular array of gold nanoseeds that is on a rotating turntable. Adjacent nanoseeds shadow each other, and thus material is only deposited on the seeds and grows in a helical pattern. The helices had a filament diameter of 70 nm and a total width of 120 nm. Both micro- and nanohelices were grown, with the nanohelices having a total length of 400 nm and the microhelices 2.5 μm. Using rotating magnetic fields generated by a triaxial Helmholtz coil, the motion of the nanohelices was both actuated and guided in HA gels. The velocity of the nanohelices was 1.1 ± 0.33 µm s^−1^ and 1.06 ± 0.46 µm s^−1^ in HA gels of 3 mg/mL and 5 mg/mL, respectively, at a magnetic rotation frequency of 50 Hz. The nanohelices also propelled in all four plane directions at a frequency of 50 Hz. Notably, when the same nanohelices were placed in glycerol/water mixtures, the nanohelices’ trajectories were not as straight. This is because, in the less-viscous liquid environment, Brownian motion exerts a greater destabilizing influence on the orientation of the helices. This study demonstrated that nanohelices can be propelled in viscoelastic gels such as HA gels and their direction can be controlled with high precision. 

Building on this work, Walker et al. [52] demonstrated that propulsion of microhelices could be achieved in mucin hydrogels supplemented with urea and bile salts (Figure 2B). Their study produced magnetic microhelices of a few µm with an 8 nm shell of alumina (Al_2_O_3_), which render them resistant to oxidation in acidic pH. Urease enzymes were decorated onto the surface of the nanohelices to catalyze the hydrolysis of urea, which results in the release of ammonia. The released ammonia caused a rise in pH of the mucin gel, which reduces the local viscosity and allows for propulsion of the microhelices. Walker et al.’s study created a mucus model that resembles the human mucosal stomach lining and demonstrated movement of the produced micropropellers at 1.4 ± 0.5 µm s^−1^ at a magnetic field strength of 10 mT and 30 Hz frequency, a higher velocity when compared to previously mentioned studies. Therefore, the use of enzyme such as urea aids in the movement of SPPs with increased velocity. Although this study used microhelices, which can limit their use in drug delivery applications due to their size, future applications may require smaller helices, which have been grown in lengths as small as 100 nm [122]. While this technique holds significant promise for achieving efficient motility in ECM, future biomedical applications will require biocompatible tracking methods for the helices, as well as methods of loading and releasing cargo.

**Figure 3:**
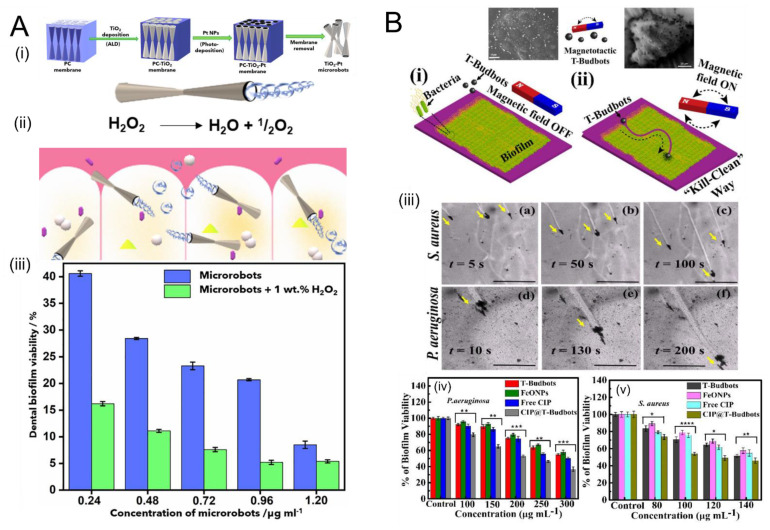
Use of self-propelled particles to disrupt bacterial biofilms. (**A**) (i) fabrication schematic for tubular titanium dioxide “microrobots” decorated with platinum (Pt) nanoparticles. The Pt nanoparticles catalyze the breakdown of hydrogen peroxide into oxygen and water, propelling the microrobots through the biofilm, which has potential applications for dental biofilms since H_2_O_2_ is widely used in this area (ii). (iii) In the presence of microrobots and H_2_O_2_, the biofilm viability is decreased compared to the case of microrobots alone (blue; no H_2_O_2_ fuel). Panel (**A**) adapted from [123]. Copyright © 2021 the authors. (**B**) (i,ii) Schematic of operation of “T-Budbots”, which are SPPs derived from natural tea plants and decorated with magnetite nanoparticles. These nanoparticles execute magnetic-driven motion through both *Staphylococcus aureus* and *Pseudomonas aeruginosa* biofilms (iii) at various time points (a–c, d–f). For both *P. aeruginosa* (iv) and *S. aureus* (v), the T-Budbots showed the most efficient reductions in biofilm viability compared to several controls (T-Budbots alone, magnetite nanoparticles alone, or free antibiotic alone). In panels (iv) and (v), statistical significance is signified by * (*p* < 0.05), ** (*p* < 0.005), *** (*p* < 0.001), and **** (*p* < 0.0001), evaluated by one-way analysis of variance (ANOVA). Panel (**B**) adapted with permission from [124]. Copyright © 2021 American Chemical Society.

Another potential application of magnetically powered SPPs is in remediation of biofilms. Biofilms are common sources of hospital infections because of the ease with which they form on surfaces and the antibiotic resistance the EPS matrix confers to the bacteria. An innovative strategy was recently demonstrated to take advantage of plant-derived “T-Budbots”, decorated with magnetite nanoparticles, to kill and clean *Pseudomonas aeruginosa* and *Staphylococcus aureus* biofilms (Figure 3B). The T-Budbots can further be attached with antibiotics, which they release preferentially at a low pH, while the drug release is minimal at a higher pH. This work shows the promise of SPPs to maneuver through ECMs in abiotic (e.g., catheters and implants) or biotic (e.g., teeth or mucosal lining) for antimicrobial purposes. Figure 3 shows a visual summary of recent efforts to use SPPs for both penetration and delivery of antibiotics in biofilms. Considering the multifaceted challenge posed by biofilms, either disrupting the ECM itself or delivering cargo directly to the bacteria may be more efficacious in terms of treating the underlying health threat posed by the biofilm. SPPs could potentially be useful for both, making this an exciting subfield to watch in the coming years.

#### 3.1.2. Ultrasound Forces

Ultrasound (US) is a common imaging modality in clinical settings because of its noninvasiveness and safe operation. However, ultrasound energy can itself be used as a propulsive energy source for the movement of SPPs. A great amount of studies have been done by Joseph Wang’s group from University of California, San Diego, involving US propelled micro/nanoswimmers. Ultrasound can lead to propulsion of SPPs in two major ways.

First, US energy can vaporize onboard hydrocarbon fuel, leading to “microbullets” or “nanobullets” with fast velocities [42]. The propulsion of the microbullets occurs due to the expansion and vaporization of perfluorohexane droplets bound within the interior of the microbullets and triggered by a US pulse. These microbullets (2.5 µm in diameter and 40 µm long) were used to penetrate tissue sections from lamb kidney. Due to rapid vaporization of the fuel, the microbullets deeply penetrated the tissue at an average velocity of 1750 µm s^−1^ and travelled 200 µm into the tissue from a single US pulse. Wang’s group also demonstrated *in vitro* delivery of cargoes such as doxorubicin, a chemotherapeutic, and silencing RNA (siRNA) using US propelled gold nanowires [125,126]. These micron-sized US sensitive nanowires demonstrated delivery in MCF-7 breast cancer cells, HeLa ovarian cancer cells and human embryonic kidney HEK293 cells. Following the fabrication of gold nanowires, Wang’s group synthesized titanium and gold nanoshells which demonstrated autonomous motion in aqueous fluid [127]. Their study demonstrated that under acoustic field, nanoshells of different diameters (ranging from 5 to 0.5 µm) have velocities that increase from 9.9 ± 1.2 µm s^−1^ to 89.9 ± 31.1 µm s^−1^. Their direction could be controlled by an external magnetic field, which allowed their internalization and directional movement in MCF-7 cells. These studies of US-propelled microswimmers/SPPs demonstrated their movement in *in vitro* cell cultures with considerable velocities. 

Wang’s group have also demonstrated cargo loading and release at acidic pH using nanorods in Garcia-Gradilla et al.’s work [128]. In their study, a polymeric segment composed of polypyrrole-polystyrene sulfonate (PPy-PSS) is introduced onto the SPPs. The positively charged dye brilliant green was electrostatically retained on the surface of negatively charged PPy-PSS. In acidic solutions of pH 4.5, the drug was released due to disruption in the electrostatic interactions. In diseases such as solid tumors, the core of the tumor is hypoxic with an acidic pH because of metabolic acidosis. The acidic microenvironment is thought to confer resistance to radio- and chemotherapy and promote cancer cell invasiveness. Bacterial bioflims also exhibit such gradients in pH. The approach of Garcia-Gradilla’s work is promising because the cargo is preferentially released in an acidic environment.

Although the above-mentioned ultrasound propulsion studies show important promise for tackling many of the challenges of navigation through ECM, the efficacy of this propulsion mechanism to propel particles through viscoelastic media, such as ECM, remains to be quantified.

To address this issue, Ahmed et al. [129] synthesized PEG-based, acoustic-powered microswimmers that propelled in a shear-thinning hydrogel. The rectangular body of the microswimmer traps air bubbles in its indentations of 50 to 100 µm in diameter and a depth of 70 µm, which is also the length of the microswimmer. The surface of the PEG microswimmers is treated to be hydrophobic, hence an air bubble is trapped in its body in hydrophilic media such as hydrogel. Movement is propelled by the acoustically driven air bubble at a velocity of 50 µm s^−1^. Hence, this study demonstrates that US-driven SPPs can propel in viscoelastic settings such as hydrogels and can be further exploited for drug delivery.

### 3.2. Remediation of Biofilms Using Chemically-Powered Motion

Although normally the reliance of conventional SPPs on hydrogen peroxide (H2O2) fuel is an impediment to their use in the body, since H_2_O_2_ is toxic. However, H_2_O_2_ is used in certain medical and dental procedures. Villa et al. [123]. made use of this fact to develop tubular H_2_O_2_-powered SPPs to penetrate and disrupt dental biofilms (Figure 3A). These SPPs, based on titanium dioxide decorated with platinum nanoparticles, were shown to penetrate dental biofilms, demonstrating a 95% bacteria killing efficiency after 5 min of treatment. They confirmed the biocompatibility of these SPPs with epidermal and organ cells. Since H_2_O_2_ is already used in dental treatments (e.g., for whitening of teeth), the reliance on this normally toxic fuel does not preclude its use in certain clinical applications. 

### 3.3. Enzymatic Propulsion

Enzymatic reactions, in which enzymes catalyze the breakdown of their respective substrate molecules, can lead to autonomous motion of particles and fluids. Colloidal particles coated asymmetrically with enzymes self-propel in solutions of the enzyme’s substrate; stationary surfaces coated with enzymes function as autonomous pumps in the presence of the substrate. In each case, the kinetic energy of the particle or fluid motion originates from chemical energy stored in the substrate molecules. Accordingly, in the last decade, enzymes have become popular as a means for driving SPPs [130] since they provide a biocompatible propulsion strategy that uses the enzyme’s substrate, which is often available in the body, as an energy source [131]. 

**Figure 4:**
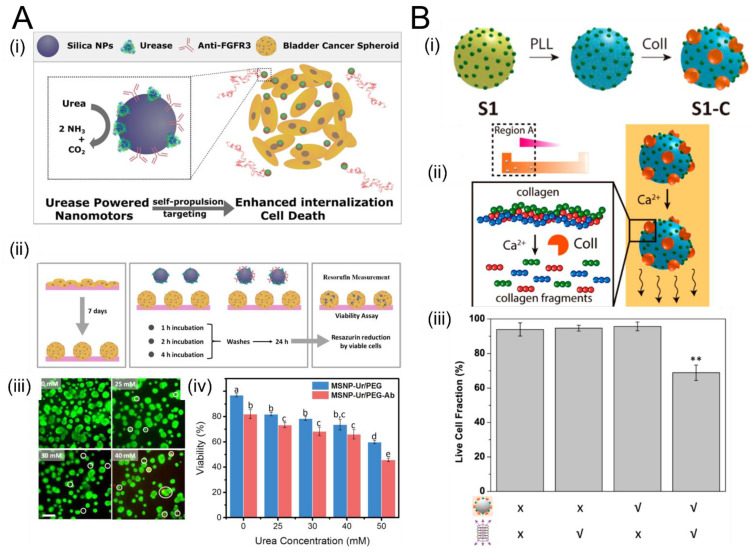
The use of enzymatically propelled particles to penetrate tissue spheroids and organoids. (**A**) (i) Schematic of using urease-decorated mesoporous silica nanoparticles (MSNPs) that self-propel in both simulated and real urine (using urea as a fuel) and penetrate bladder cancer spheroids. (ii) Experimental methodology for growing spheroids over 7 days, incorporating MSNPs, and assessing spheroid viability. (iii) Live/dead assay of spheroids after 4 h of incubation with MSNPs at four concentrations of urea fuel: 0, 25, 30, and 40 mM (scale bar 200 μm). (iv)Quantification of spheroids’ viability after 4 h of incubation with MSNPs functionalized with anti-FGFR3 antibodies (red) versus MSNPs without antibodies (blue) at different urea concentrations; different letters (a through e) above the bars denote significant differences among groups with *p* < 0.05, N = 3. Panel (**A**) adapted from [140] Copyright © 2021 American Chemical Society. (B) Manganese ferrite (MF-NP) microswimmers were developed based on collagenase that penetrated human osteosarcoma spheroids and enabled localized hyperthermia-based treatment using magnetic fields. (i) Addition of collagenase (Coll) to polystyrene nanospheres coated with one layer of magnetic material (S1) and an intermediate poly-L-lysine (PLL) layer. (ii) In the presence of calcium ions (Ca2^+^), the MF-NP swimmers move by breaking collagen fibers down into smaller fragments. (iii) Fraction of live cells in the organoid in the presence and absence of MF-NP swimmers and alternating magnetic fields (AMF). Live cell fraction is significantly decreased in the presence of both MF-NP and AMF (rightmost bar) compared to the case when either or both are absent. This work demonstrates the efficacy of enzymatic SPPs for advanced cancer hyperthermia treatments. ** indicates *p* < 0.01, as determined using a one-way ANOVA followed by Tukey’s multiple comparison *post hoc* test. Panel (**B**) adapted from [105] Copyright © 2021 American Chemical Society.

Figure 4 shows salient examples demonstrating enzyme-driven propulsion of SPPs in ECM-like microenvironments. Hortelão et al. used urease-decorated mesoporous silica nanoparticles (MSNPs), which undergo urea-fueled self-propulsion in both simulated and real urine and penetrate bladder cancer spheroids (Figure 4A). This study is an important proof of principle because it demonstrates that urease generates sufficient forces to penetrate ECM-like microenvironments, leading to potential application in bladder cancer treatment. In another exciting study, Ramos-Docampo et al. developed manganese ferrite (MF-NP) microswimmers with collagenase “engines” that penetrated human osteosarcoma spheroids and enabled localized hyperthermia-based treatment using magnetic fields (Figure 4B). In the presence of calcium ions (Ca^2+^), the MF-NP swimmers move by breaking collagen fibers down into smaller fragments. As shown in panel (iii), the fraction of live cells in the organoid decreased in the presence of MF-NP swimmers and alternating magnetic fields (AMF), compared to the case when either MF-NP or AMF are absent (or when both are absent). This study demonstrates the efficacy of enzymatic SPPs for advanced cancer hyperthermia treatments.

Several types of enzymes have been demonstrated to be efficacious for propulsion. The first demonstrations of enzymatic propulsion employed a combination of glucose oxidase and catalase [132,133]. Glucose oxidase catalyzes the oxidation of glucose into D-glucono-δ-lactone and hydrogen peroxide (H_2_O_2_), and catalase further breaks H_2_O_2_ down into oxygen and water. Other common examples include urease, acetylcholinesterase, and aldolase [130,134], whose respective substrates are urea, acetylcholine, and fructose-1,6-biphosphate. 

The physical mechanism underlying enzymatic propulsion remains incompletely understood. Although the exothermicity of many enzymatic reactions was thought to lead to propulsion in some instances, the small temperature changes involved are generally considered to not be significant enough to cause significant propulsive forces. Currently, leading hypotheses to describe the physics underlying enzymatic propulsion include diffusiophoresis and conformational changes. Diffusiophoresis refers to transport induced by a gradient in chemical concentration, in this case in the reactants and products resulting from the enzymatic reactions, which is thought to lead to a propulsive force via varying the interaction strengths between the SPP and the reactants and products [135]. However, the strength of the propulsive forces generated due to diffusiophoresis has been called into question [131]. On the other hand, conformational changes are known to occur in many enzymatic reactions, such as those involving aldolase enzymes [136], which in turn may agitate the surrounding fluid in such a way as to induce motion upon substrate binding and unbinding [137,138]. 

Enzyme propulsion has many advantages for applications in penetrating ECM. First and foremost, enzymes make use of readily available fuel that is often present in ECM (e.g., glucose, urea). Second, enzymes also can undergo chemotaxis in gradients of their substrates [139], leading to the possibility of chemotactic enzymatic propellers that exploit the many gradients available in tissue ECM as well as biofilms. Third, enzymatic-driven particles have been shown to move in ECM-like environments, leading to cell death in bladder cancer spheroids and offering a novel method for cancer thermal therapy [105,140]. 

Further research will be necessary to understand more fully the physics underlying enzyme-mediated propulsion, so that we may better understand and exploit their capabilities for navigation and cargo delivery in diseased ECM environments. This will enable the optimization of the design of enzymatic particles.

**Table 1:** Summary of the different SPPs as well as the advantages and disadvantages of their mode of propulsion.

SPP Design	Reference	Type of Propulsion	Advantages	Disadvantages
Ferromagnetic nanorods	Mair and Superfine [51]	Magnetophoresis in static magnetic field	Simple fabrication and operation No complex set-up required for propulsion or steering	Slow No data on biocompatibility
Nano-/microhelices	Mark et al. [122] Schamel et al. [50] Walker et al. [52]	Rotating magnetic field	Efficient movement in viscoelastic media	Complicated apparatus required to generate rotating magnetic field
			Thin helical filaments maneuver through narrow ECM mesh	No data on biocompatibility Cargo delivery not trivial
Rectangular propellers	Ahmed et al. [129]	Ultrasound/Acoustic powered	Synthesized from polymers which are biocompatible Can achieve higher velocities than magnetically propelled SPPs.	Size of the propellers can be a hindrance in delivery
Micro-/Nanobullets	Kagan et al. [42] Soto et al. [141]	Ultrasound-induced vaporization of onboard hydrocarbon fuel	Extremely high speeds and strong propulsive forces Demonstrated to penetrate lamb kidney tissue	Challenges controlling initial, final locations, motion direction Biocompatibility of materials and fuels unclear Single-shot implementation
Enzymatic Particles	Hortelão et al. [140] Ramos-Docampo et al. [105]	Enzymatic reactions	Use readily-available biological fuels (e.g., urea, glucose) Enzymes undergo chemotaxis, potentially allowing “smart” propelled nanocarriers	Patterning enzymes precisely is challenging Scaffolds used (e.g., silica) are not biocompatible Propulsion mechanism not fully understood

## 4. Summary and Outlook

Autonomously propelled micro- and nanoparticles hold tremendous promise for treatment of ECM-mediated diseases and remediation of bacterial biofilms. As discussed herein, researchers are developing new strategies to generate propulsive forces that efficiently penetrate extracellular matrices of various types as well as release cargo at controlled rates and in specific locations, improving the delivery efficiency over conventional active or passive nanocarriers. 

Future research must focus on translating the exciting advances reported herein, which are overwhelmingly confined to research laboratories, into clinical therapies that help achieve better patient outcomes. Toward this goal, we close this article with several suggestions for future research directions:

1. *Effect of ECM mechanics and rheology on propulsion efficiency*: The motion efficiency (and thus therapeutic efficacy) of SPPs has been shown recently to depend on the topology, rheological properties, and viscoelastic properties of the medium. Recently, it was reported that shear-thinning effects cause a substantial enhancement in the propulsion of helical microswimmers [114]. On the other hand, it is also known that as ECM becomes stiffer and denser, the velocity is attenuated. Thus, the behavior of a given SPP design in a given ECM is not necessarily trivial to predict *a priori*.
2. *Effect of SPP propulsion mechanism, size, and shape on propulsion efficiency*: Since the trends are likely to depend strongly on the design of SPP under consideration, systematic analyses with multidimensional parameter spaces (e.g., ECM stiffness, ECM viscoelasticity, porosity, etc.) may be necessary for individual SPP designs.
3. *Testing in biomimetic materials*: As alluded to in #1, the evidence increasingly shows that SPP performance depends strongly on the mechanics, viscoelastic properties, and topology of the ECM (as does cell migration through these same matrices). As a result, while they may be an important first step in verifying propulsion, drug delivery experiments conducted in a simple Petri dish are unlikely to be indicative of future therapeutic potential. Instead, it will be critical that future *in vitro* studies of SPP performance be conducted in media that accurately recapitulate the mechanical and rheological properties of the tissues in which they are to be used. Vigorous research is underway toward the development of novel, tunable biomaterials that closely resemble those of in vivo tissues. Interdisciplinary collaborations between these groups will be crucial to this stage of development.
4. *Advanced SPP design methodologies*: The size, shape, and propulsion mechanism of SPPs clearly affect their performance in ECMs. With different ECM biochemistry, material properties, porosity, and mechanical and rheological properties, new designs for SPPs may need to be invented. With the advent of advanced manufacturing techniques such as micro- and nanoscale 3D printing, as well as the continued growth of nanofabrication techniques, it may soon become possible for creative researchers to design SPPs with bespoke shapes for a given ECM application.
5. *Tracking*: Although it was not the focus of this review, a crucial component of successful translation of SPP-based therapies to the clinic will require the development of clinically-compatible tracking methodologies. Exciting progress has been made in this area recently with the use of photoacoustic computed tomography (PACT) that enables tracking of magnesium-based SPPs in the digestive tract of animal models [142]. However, the performance of many of these tracking methods within tissues has yet to be demonstrated quantitatively.
6. *Theoretical and simulation studies*: Although this review has focused on experimental demonstrations of SPPs moving in ECM models (and, in some limited cases, in vivo ECM), we wish to emphasize the importance of theoretical investigations to the design of SPPs with optimal properties to maximize the efficiency of motion through ECM. A recent study exemplifies the promise of this approach. Aceves-Sanchez et al. [143] theoretically studied the collective motion in an environment filled with spheres tethered to fixed points in space via linear springs, which play the role of obstacles (such as ECM fibers). They showed that this obstacle-based environment can induce aggregation of SPPs. As they and others have noted [144], aggregation is known to correlate with the ability of metastasizing cancer cells to migrate; by the same token, aggregation should be taken into account when designing future SPP-based therapies, in which it could serve as both a hindrance (e.g., if it stops the motion entirely through steric interactions) or a help (if it allows more cargo to be transported while still permitting motion). Going forward, a close coupling between theory and experiments will be crucial to converge on the most efficacious designs.

Development of SPPs is inherently interdisciplinary and distributed throughout laboratories around the world. With interdisciplinary collaboration among chemical engineers, bioengineers, materials scientists, radiologists, and oncologists, SPPs could be viable for clinical trials within the coming decade or sooner.
